# Supplementary material for: Multiple nest entrances alter foraging and information transfer in ants
Source: R Soc Open Sci. 2020 Feb 26;7(2):191330. doi: 10.1098/rsos.191330 (PMC7062076; doi:10.1098/rsos.191330)
Supplement: Asymmetry index of entrance use [file rsos191330supp1.docx]

| **Table S1**. Asymmetry index of entrance use | | |
| --- | --- | --- |
|  | asymmetry index | |
| colony | after 5 min | after 120 min |
| 1 | 0.27 | 0.05 |
| 2 | 0.56 | 0.17 |
| 3 | 0.37 | 0.10 |
| 4 | 0.20 | 0.08 |
| 5 | 0.53 | 0.02 |
| 6 | 0.33 | 0.13 |
| 7 | 0 | 0.04 |
| 8 | 1 | 0.32 |
| 9 | 0.67 | 0.43 |
