# Supplementary material for: Multiple nest entrances alter foraging and information transfer in ants
Source: R Soc Open Sci. 2020 Feb 26;7(2):191330. doi: 10.1098/rsos.191330 (PMC7062076; doi:10.1098/rsos.191330)
Supplement: Trail Detection [file rsos191330supp2.docx]

Trail Detection (See figure S1 for a visual presentation)


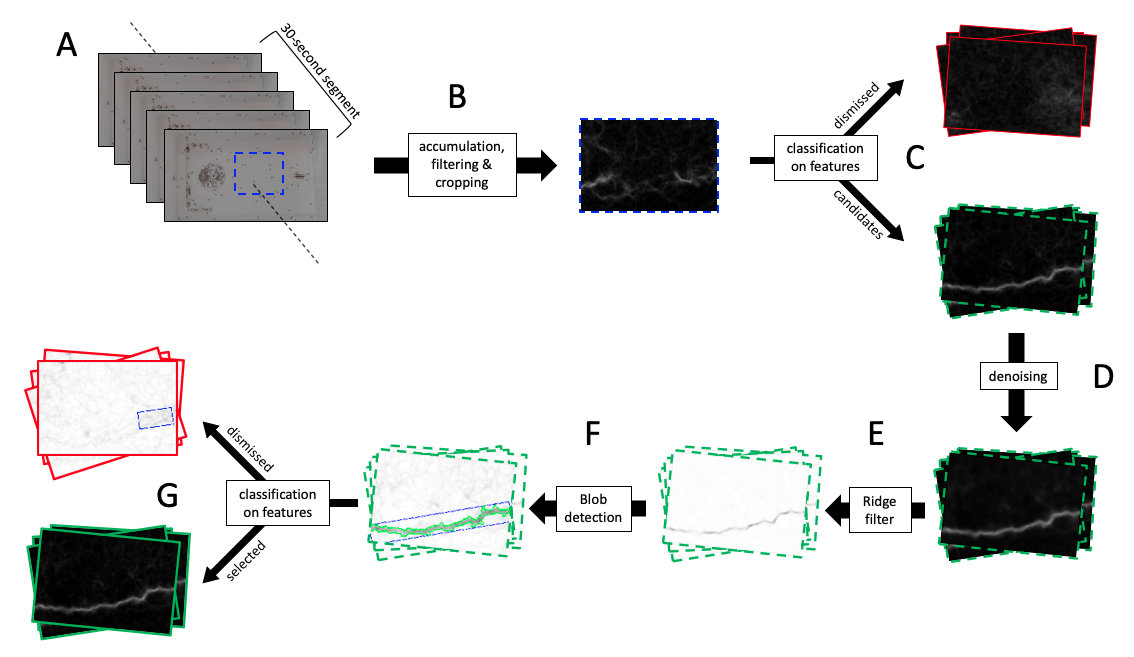


**Fig. S1**. Algorithm used to detect the formation of a common foraging trail. (A) A region of interest (ROI) is cropped out in each frame of the video stream (in blue dotted line). Then, ants are detected in this ROI by a motion-detector based on background subtraction. (B) The detected mobile pixels are then overlaid over 5-minutes period into a single frame for a total of 24 frames per experiment. (C) A first decision tree based on image features is used to classify the 2 * 9 * 24 = 432 frames into “dismissed” or potential “candidates”. (D) The potential candidates are denoised with a Gaussian filter. (E) The algorithm applies a ridge filter to highlight potential ridges on the resulting images. (F) The ridges are detected by a blob detection method. (G) The candidates are then sorted into “dismissed” or “selected” by a decision tree based on the features of the largest blob detected on the image.

For each experimental video, frames were read from the video stream and converted from RGB to grayscale. For each frame, we cropped a 160 x 240 pixels area located between the nest and the food source. Then, we subtracted a background image in order to retain only the pixels in motion, corresponding to the moving ants. The background was computed as a running average of the previous frames. By doing so, the background was dynamically adjusted so that motionless objects became gradually part of the background while moving objects were not integrated. After subtracting the background, the resulting grey-scale image was thresholded and converted into a black and white binary image. Finally, binary images were stacked over 5-minute-periods in order to overlap images of all detected moving ants. To do so, we summed the binary image and remapped the image matrix into the interval [0; 255] to obtain a grayscale image. This resulted in 24 successive images for each experiment and a total of 432 images for all experiments.

The following decision tree was used to classify images into “dismissed” or potential “candidate”. These images were first sorted out according the spatial distribution of their pixels. In particular, we used the total intensity of the image, the distribution of this intensity along the vertical and horizontal axis, and the entropy of the image computed as:

$$H=-\sum_{i=0}^{n-1} p_{i}\log p_{i}$$

with *n* the number of potential grey levels forming the image, and *p_i_* the probability of a pixel having the grey level *i*.

A strong recruitment of foragers would be associated with a high intensity of the pixels as many movements are detected. Then, in the ideal case of a trail connecting the nest entrance(s) directly to the food source, we would get a horizontal beeline structure concentrating most of the intense pixels. Such pattern would be associated with a high heterogeneity of the pixels’ intensity on the vertical axis (with a higher intensity near the trail) and a low heterogeneity of the pixel intensity along the horizontal axis. Finally, a concentration of the activity along a single trail would result in a rather bimodal distribution of the pixel intensity with the majority of pixels with a low intensity and small number of pixels with a high intensity forming the trail. On the contrary, random movements of the ants would lead to a more homogeneous distribution of the intensity among the pixels. The detection of a strong trail would thus be associated with a low entropy while random walks of foragers would be associated to a higher entropy of pixels’ intensity. Ultimately, potential candidates were selected by the algorithm as images with a high overall intensity distributed homogeneously along the horizontal axis and heterogeneously along the vertical axis, and associated with a low entropy.

These candidates were then denoised by a gaussian filter, then a ridge filter. Finally, a blob detector was used to identify the largest blob on each resulting image. The candidates were then sorted by a decision tree based on the size of this blob, its ratio between the minor and major axis of the blob and the orientation of its major axis. The algorithm detected as “trail” blob that were sufficiently large, elongated and oriented on the left-right axis and dismissed other images.

To determine the different parameter’s values of the algorithm, each author sorted independently the 432 images by eye into three different classes: (i) Clear-cut presence of a trail, (ii) Clear-cut absence of a trail, (iii) Undetermined. Then, we built a ground truth dataset by selecting only the images that were labelled with a clear-cut presence or clear-cut absence of a trail by the three authors. The threshold values used for first algorithmic classification based on the image characteristics were chosen to reduce the number of false negative detections to zero (i.e. all images that were classified as “Clear presence of a trail” by the authors had to be detected by the algorithm during the first classification step). Then, we evaluated the efficiency of the second step of the algorithm (ridge detection) by trying several ranges of parameter’s values and computing the confusion matrix of each set of values. We selected the parameter’s values that minimized the total number of errors (false positive + false negative). The algorithm was then run on the 432 images and the resulting classification was considered as final.

**Fig.S2** *Images representing the cumulated activity detected in the foraging area for 5-minute periods over the 120 min experiment.* The nine top rows represent the experiments with one-entrance nest while the nine bottom rows represent the experiments with two-entrance nests. Periods of “structured foraging” are highlighted in green while periods of “unstructured foraging” are highlighted in red.
